# The Use of Sonification for the Analysis and Teaching of Interpretive Auditory Nuances

**DOI:** 10.3389/fnins.2022.808038

**Published:** 2022-03-17

**Authors:** Kathleen Riley

**Affiliations:** Department of Music, Steinhardt School of Culture, Education, and Human Development, New York University, New York, NY, United States

**Keywords:** sonification, Disklavier, MIDI, interpretive nuance, imitation

## Abstract

The integrative powers of human auditory perception in masterful performing artists can create nuances of sound from the interpretation of notes on a written page that often seem beyond one’s grasp as a listener. It is important to consider what type of feedback can provide a clearer understanding of nuances in sound to guide motor learning for the acquisition of new skills for expression. Master pianists’ performance has been used as a model for imitation. However, to ensure the accuracy of imitation and clear understanding, sonification was examined for its effectiveness in providing a more immediate understanding of individual interpretations in terms of inter-onset timing and velocity of the notes. Three master concert pianists volunteered to record a performance of the Chopin Nocturne Opus 15 No. 1 on a Yamaha Disklavier Pro MIDI (music instrument digital interface) grand piano. Logic software was used to analyze and compare MIDI data from each performance from the perspective of phrase-by-phrase, note onset timings, and corresponding data from the other pianists. The study objectives were to examine commonalities and differences in timing and dynamics among the performances using MIDI measurements; to probe whether listening to comparative performance data assisted with feedback from sonification would enhance music students’ understanding of the interpretive nuances through imitation; and to determine whether auditory-assisted sonification feedback could be used as a tool to expand students’ interpretive choices and enhance performance. Participants imitated selected phrases of each of the master pianists, first with only music listening, and then with feedback from the sonified, comparative performance data. Results showed limited success in attempts to imitate the model with auditory feedback alone. Auditory-assisted sonification feedback significantly enhanced the participants’ abilities to imitate the model faster and with greater accuracy in the final imitation experiments. The data gathered by the study provide insights on this kind of sonification as an effective feedback tool for heightening participants’ auditory understanding of nuances in sound, as well as providing an effective teaching tool for imitation exercises.

## Introduction

The integrative powers of human auditory perception are capable of creating highly nuanced experiences of sound through inflection, modulation, and resonance. Musical performances, whether heard live or recorded, provide excellent opportunities to investigate such powers. Moreover, the study of interpretive nuances produced by master performing artists provides an even greater opportunity to explore nuances that are normally intangible.

Despite the widespread assumption that musical scores could also be useful in this regard, sonification, that is, the visual representation of notes on a music score, cannot convey the complexity of such nuances and their infinite possibilities of variation. The parameters of interpretive expression in performance have been thoroughly investigated and analyzed in previous studies (Sloboda, 1983, 1999; Shaffer and Todd, 1987; Clarke, 1988, 1989, 1993; Gabrielsson, 1988; Palmer, 1989, 1996; Repp, 1992, 1995). Studies have also examined pianists’ individuality in performance (Seashore, 1936, 1938; Bernays and Traube, 2014).

The purpose of this exploratory study was to investigate whether the use of information (data) from the musical instrument digital interface (MIDI) of artists’ performances for a comparative analysis would improve piano students’ understanding of the differences among the interpretive techniques used by performers. The Chopin Nocturne Opus 15 No. 1 was selected as the composition to be performed and recorded. In addition, the study investigated whether these interpretive differences could be reproduced by students for selected phrases of the artists’ performances as part of their process of learning a composition.

To analyze the interpretive nuances in the artists’ performances, an auditory-assisted sonification of the MIDI data was examined, presented as a performance score. The printed score analysis symbols served as coded instructions for the performers to recreate the sounds intended by the score. The sound data received as MIDI allowed the performance to be played back on the instrument itself, *via* music sequencing software. MIDI data provided real-time sonification (piano roll) during the playback, allowing a simultaneous audio and visual analysis (Dombois and Eckel, 2011).

Such simultaneity enables sounds that are produced in time and are transient, as opposed to static visual displays that are repeatedly heard and examined by the observer as sound in time (Bonebright and Flowers, 2011). Auditory and visual feedback have been shown to be effective in controlling pitch production by instrumentalists, singers, and speakers (De Bot, 1983; Blanco et al., 2021). As auditory percepts influence our visual percepts, visual percepts may in turn influence the perception of an auditory representation (Neuhoff, 2011). Previous research has shown that enhanced visual feedback can have remarkable effects on the acquisition of technical skills (Tucker et al., 1977).

The sonification graph displays detailed measurements of the properties of time, space, and velocity information. Playback of the data shown in the graph was performed simultaneously using Disklavier. This factor is important in understanding the use of sonification in this study, as it provides a sonification of a “sonification” (the notated score) as interpreted by different performers.

The study also investigated whether auditory-assisted sonification can increase the accuracy of students’ model imitations by providing feedback on the extent to which the imitation does or does not match the model. Humans learn by imitating models, as demonstrated in the visual arts, drawing, painting, and sculpting. Implicit learning is based on the notion that cognitive processing is entirely unconscious (Berry and Dienes, 1993). A significant benefit of teaching through modeling and imitation is that students’ listening and evaluation skills improve as they attempt to match the model (Haston, 2007).

## Materials and Methods

### Participants

Three concert pianists participated in this study, namely, Sara Davis Beuchner, Garrick Ohlsson, and Gerald Robbins, along with eight college undergraduate piano majors from New York University.

### Analytical Tools

**FIGURE 1:**
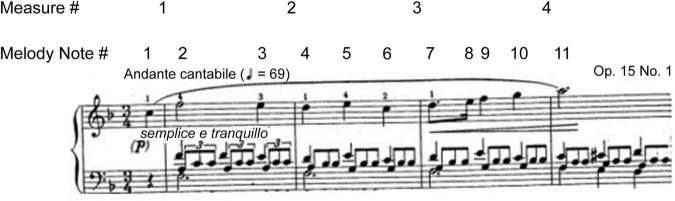
Measures 1–4 from the piano score.

The Disklavier Pro Piano, the Disklavier GranTouch (a non-acoustic instrument), a MIDI interface, and logic music editing software were used to record data and play back all performances. The setup of the experiment is shown in Figure 1.

#### Musical Instrument Digital Interface

The MIDI is a data interface that conveys musical messages. MIDI instruments describe performance variables by reading and outputting a constant stream of messages. The velocity of each note is represented by numbers, notes are identified and placed in the correct octave, and the duration of each note is recorded.

The MIDI data must flow through a sound-generating device. A MIDI interface is used to communicate with a sequencer or another device. In this study, the devices are the Logic sequencer software, the Yamaha Disklavier Pro, and the Yamaha GranTouch. After the MIDI data are fed into these sound generating devices *via* cables, they can be perceived as audible sounds.

#### The Disklavier

The Yamaha Disklavier Pro was used to record all performances. Originally using floppy disks as the recording medium, the digital device plays an acoustic piano during playback, ensuring that tonal irregularities, noise, and distortion typical of piano audio recordings are avoided. Pro has a wide range of MIDI features that allow recording and playback with an external MIDI sequencer, such as Logic 4.0. However, Disklavier Pro has a 500 ms delay in production due to the mechanics of the solenoids driving hammers.

Therefore, Disklavier GranTouch (a non-acoustic piano) was chosen for the initial comparative analysis of the performance’s timing. The advantage was the absence of a delay between seeing the sonification of the notes as piano roll bars on the screen and hearing the sound produced.

#### Logic Sequencing Software

Logic software was originally used to analyze the MIDI data, and Logic Pro was used to update and edit the sonification diagrams. The performances were not recorded to the beat of a metronome, but were interpreted freely and expressively. To compare precise measurements of timing in the spontaneous performances, the piano roll editor was used, in which the timing is displayed as Society of Motion Picture and Television Engineers (SMPTE) code. SMPTE provides a unique address for each frame of a video signal. The address is an eight-digit number representing hours, minutes, seconds, and frames. In the phrase-by-phrase analysis, seconds and frames were used for the measurements and comparison. Each performance was analyzed and compared to the phrase and note onset times, and compared to the corresponding data from the other pianists. Tempo changes in phrases were calculated from differences between onset times of successive notes. The piano roll editor in the Logic software provided a graphical representation of the physical parameters: timing and dynamics of the performance, specifically, note duration, inter-onset interval (IOI) timing, note overlap, and velocity. The recorded data of the performances were heard by playing them back on the Disklavier, while they were displayed as sonifications in real time. Figure 1 shows the notated scores of measures 1–4 of the Nocturne.

**FIGURE 2:**
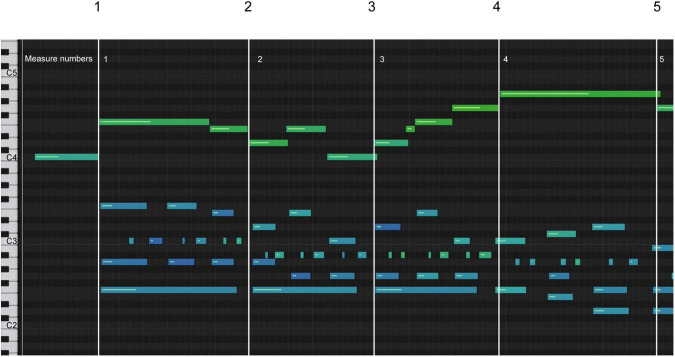
Sonification graph of measures 1–4.

Figure 2 shows a sonification graph. The keyboard is displayed on the left side of the diagram. The horizontal bars represent the notes and their durations. The velocity range of 0–127 is represented by a color spectrum of the note bars: purple (quietest) >blue >green >yellow >orange >red (loudest). White lines have been added to represent the measurement lines. The actual velocity number, representing the dynamic level of each note, was determined in the event editor for the data analysis.

### Procedure

Three concert pianists recorded Chopin Nocturne Opus 15 No. 1 on the same Disklavier Pro. Section A of the Nocturne was selected for a comparative analysis of the three performers.

Eight college piano students volunteered to participate in the study. The level of note reading and musicianship varied. None of the participants had studied the Nocturne before. They participated in three performance analysis sessions and imitation trials with auditory-only feedback as well as auditory-assisted sonification feedback.

The student performances of Section A of the Nocturne were recorded on the Disklavier at the beginning, middle, and end of the study. As a baseline condition, each participant recorded the Nocturne before listening to the performers (henceforth referred to as the “model” performances). All eight were at different stages of musical and technical development.

The study was conducted in three phases:

1. Comparative performance analysis of the model performances.
2. Presentation of the performance analyses to the students
  (a) Auditory playback with notated score
  (b) Simultaneous auditory playback with sonification score.
3. Imitation of selected phrases of the model performances, improvement of technical mastery and auditory comprehension.
  (a) Improvement of technical mastery and auditory comprehension.

In Phase 2, students’ auditory perception and technical skills were assessed with a series of questions after listening to the performance for the first time without visual feedback from the sonification graphic. The following questions were asked:

(1) Do all of the performances follow the dynamic markings on the score?
(2) Which performer had the greatest range in dynamics?
(3) Where does Pianist A relax the tempo in the first phrase? Pianist B? Pianist C?

Students were then presented with a comparative performance analysis of the models. Auditory-assisted sonification was used for the analysis.

Selected phrases from the models were chosen for imitation, first with the listener alone, then with the auditory-aided sonification. Auditory-assisted sonification provided immediate feedback on the extent to which each imitation matched the model. For data analysis, the MIDI data of the timing and speed of the imitations were compared with the data of the selected model.

#### Treatment of Students

During the comparative analysis and the imitation experiments with feedback, sonification was visible on the computer screen, while the performances on the Disklavier were heard in their entirety and in sections.

**FIGURE 3:**
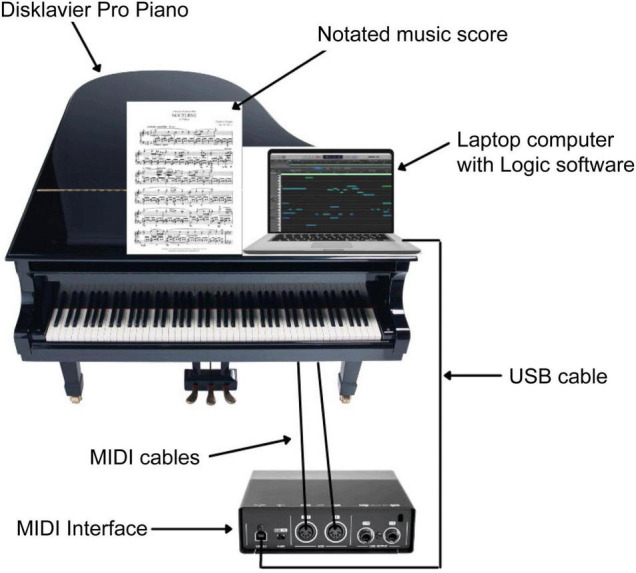
Schematic drawing of the setup.

The computer with the Logic software was positioned on top of the piano to the right of the music stand and connected to the MIDI interface *via* a USB cable. The MIDI interface was connected to the Disklavier *via* the MIDI cables (Figure 3). The 500 ms sound delay during MIDI playback by Logic was disabled on the Disklavier for simultaneous acoustic/visual analysis and for the imitation experiments with auditory-assisted sonification. The delay was re-enabled for the faithful reproduction of the velocities while listening to and comparing the performances and for the imitation experiments. For the experiments conducted without feedback, the performances and selected phrases were played back from a CD.

All readings and imitation experiments were performed and recorded on the Disklavier Pro. The data from MIDI were analyzed using velocity numbers for dynamics and SMPTE time codes for note onset in the event editor. Sessions were tape-recorded to capture the participants’ responses.

## Phase 1: Comparative Performance Analysis

The diagrams of sonification performance showed interpretive deviations from the regularity of the score information in terms of note duration, intensity, and articulation. Analysis of MIDI data allowed quantification of spontaneity in terms of timing and dynamics. SMPTE data were analyzed in terms of note duration, IOI, note overlap, and velocity. Auditory-assisted sonification diagrams revealed similarities and differences in the dynamics and timing of the performances by the three artists of Section A of the Nocturne. Sara Davis Beuchner’s audio/video sonification, referred to in this article as Model A, is included as Sonification 1; Gerald Robbins as Model B, Sonification 2; and Garrick Ohlsson as Model C, Sonification 3.

Subsequently, the sonification diagrams of each model’s performance on measures 1–4 are presented with an analytical discussion focusing primarily on rhythm and examining the neuromuscular responses of the fingers to auditory intentional cues. Sonification files 1, 2, and 3 can be used for auditory/visual sonification.

**FIGURE 4:**
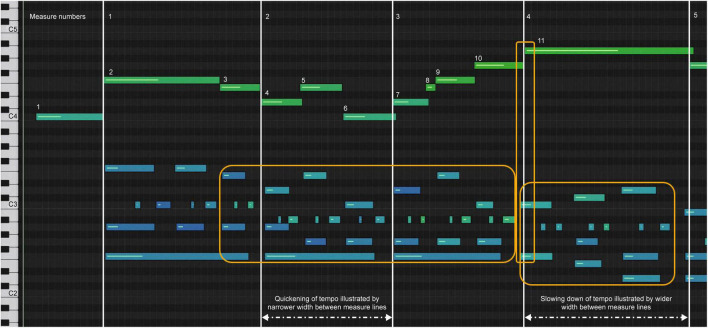
Sonification score, model A, measures 1–4.

Model A increased tempo after the downbeat in measure 1 (melody notes 3–10), indicated by the shortening of the note bar length in the melody and the length and space in the accompaniment highlighted by the yellow box in measures 3–4 (Figure 4). A slower tempo marks the end of the phrase in measure 4, indicated by the lengthening and spacing between the note bars in the left-hand accompaniment, highlighted by the yellow boxes. The end of the phrase, melody note 11, was played after the left-hand accompaniment, highlighted by the vertical yellow box around bar line 4. In Figure 1, one can see the juxtaposition of the left- and right-hand notes. Experience of audio/video sonification for Model A was provided by Sonification 1.

**FIGURE 5:**
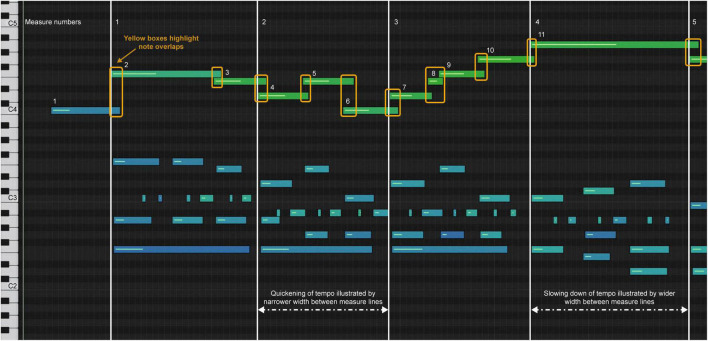
Sonification score, model B, measures 1–4.

Model B had a more regular pulse during measures 1–3, as indicated in the sonification diagram by the length of the note bars (Figure 5). The note bars highlighted by yellow boxes indicate the overlap of onset and offset. This technique was intentionally used by the performer to create legato, a musical performance technique that produces fluid and continuous movement between notes. In A and C, the overlap was not used; rather, the legato was created by the smooth transition of onset and offset between notes. Experience of audio/video sonification for model B was provided by Sonification 2.

**FIGURE 6:**
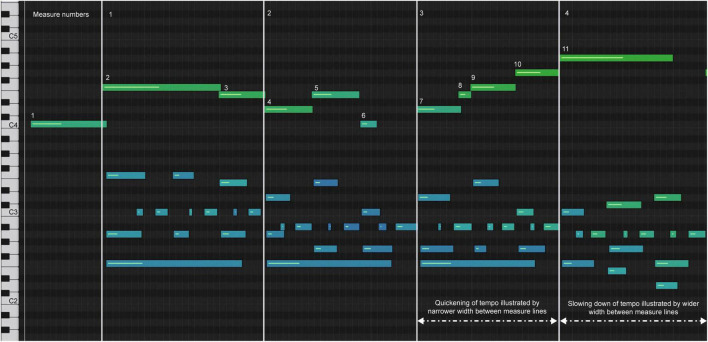
Sonification score, model C, measures 1–4.

**FIGURE 7:**
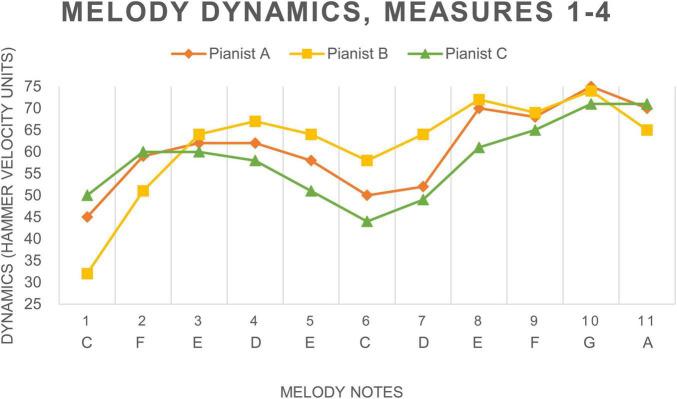
Comparison of artists’ velocity (dynamics) melody, measures 1–4.

Model C has a more deliberate opening movement and maintains a more even tempo (Figure 6), but uses a wider range of dynamics (Figure 7). The decrease in dynamics in the middle of bar 2 was greater (Figure 7, notes 5–6). The increase in dynamics that accompanied the second part of the phrase in bar 3 increased (Figure 7, notes 7–10) before decreasing slightly at note 11, which marked the end of the phrase. The beginning of the melody led slightly to the end of the phrase on each beat, which was difficult to see because of the size of Figure 6. Experience of audio/video sonification for model C was provided by Sonification 3.

All three artists began the opening phrase with an extended melody note 1 leading to note 2 (see Figures 4–6). All three marked the middle of the phrase with a decrease in dynamics (Figure 7, note 6) and a delay in timing represented by the note bar length (Figures 4–6). There was little difference between the performers in terms of the rubato and dynamic form during the phrase.

### Results of the Comparative Performance Analysis

#### Similarities

First, we found that a large proportion of timing deceleration occurs at the phrase boundaries. These are defined by harmonic progressions, most readily understood by referencing Sonification 1, 2, and 3. Second, the more the tempo slowed down, the more quietly the notes were played. All three models accompanied the timing deceleration at the section end with a decrease in dynamics. Third, there was a greater increase in velocity (dynamics) at the melodic contour peaks and lower intensities at the contour valleys. Figure 7 depicts one of these (note 6). The ascending melodic lines were accompanied by an increase in velocity.

Auditory-assisted sonification provided insight into how rhythms were produced, perceived, imagined, and expressed as the movement of sound in time. These aspects included emphasis on phrase beginnings and endings, climaxes, and cadences, such as temporal synchronizations and speed increases and decreases.

#### Differences

The differences in the overall timing of performance were as follows: model A, 1:17 min, model B, 1:24 min, and model C, 1:32 min.

Phrase-by-phrase analyses highlighted the differences and some “signature” devices of the models. Model A favored delaying the onset of the melody note for phrase beginnings and melodic climaxes. Model B built a crescendo to the penultimate note of a phrase, especially at peaks, and emphasized arrival by drop-in intensity. Model C used asynchronies between melody and accompaniment (right and left hand) to create rubatos.

Model A delayed the onset of the first melody note of the phrase in five out of six cases. Models B and C began each phrase with synchronized hands or a slight lead of the melody note. Model C paused before beginning a phrase. Model A delayed the beginning of the melody note at the end in half of the phrases. Model B rounded out the end of each phrase with a decrease in dynamics. Model C consistently slowed the tempo at the end of a phrase. The frequency and extent of deviation varied between pianists.

Implications for the educational use of auditory sonification emerged directly from the comparative analysis process. The origins of sonification as a performance score go back to the work of Seashore (1936, 1938). “After examination of the facts, the reader may be ready to sit down and attempt to reproduce one of the performance scores on the piano, interrupting the performance from point to point to “hear out” the significance of a particular variant in the phrasing” (Seashore, 1938, 242).

## Phase Two: Auditory-Assisted Sonification Feedback With Imitation as a Teaching Tool

The second hypothesis was that, with auditory-assisted sonification feedback for their imitation attempts, participants would show increasing accuracy in: (1) detecting subtle differences in the artists’ performances of the score and (2) successfully imitating the selected phrases of the performers. The following are the applications and results of the experiments.

### Imitation

**FIGURE 8:**
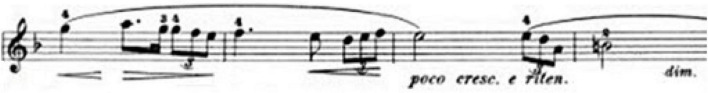
Measure 5 to downbeat of measure 8 from piano score.

Auditory-assisted sonification helped students immediately see and hear the extent to which their imitations matched the model. Imitation exercises were conducted: Melody-to-Melody and Melody-to-Accompaniment. Below is an example of a performed melody-to-melody imitation with feedback on measures 5–8 (Figure 8).

**FIGURE 9:**
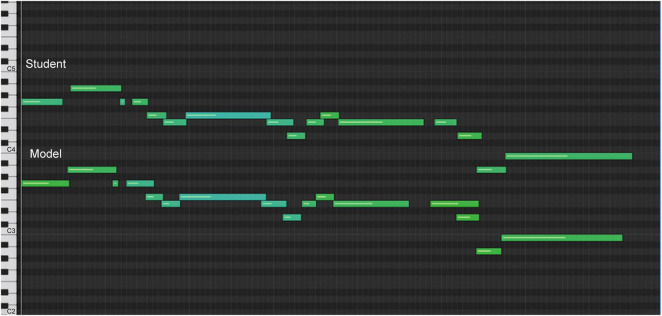
Sonification graph of imitation with feedback.

The diagram in Figure 9 shows the degree of accuracy achieved by a student on their second attempt at melodic imitation of the model. The student’s imitation is represented by the note bars in the upper part of the diagram; the model is shown below. To experience this sonification, please refer to Sonification 4.

#### Results

Repeated imitations of the melody with auditory feedback helped students become more familiar with the score. Students who exhibited stronger eye-hand coordination achieved optimal imitation of timing (0–0.05% discrepancy) after fewer repetitions. Those who exhibited weaker reading and music skills became more familiar with the sonification score through repeated imitations. The degree of discrepancy decreased, and they achieved optimal imitations (0–0.05%).

The optimal imitation of the melody for accompaniment with feedback was achieved within two to four repetitions. Once this was achieved, the students were able to consistently maintain the optimal level with feedback. The degree of discrepancy in timing decreased with each session, while the difficulty of the phrases increased.

## Discussion

The sonification diagram displays specific details of how a pianist interacts with the instrument to create complex and expressive sounds in terms of timing and speed. Critical attributes that shape and define the musical instrument are the interaction of coordinated hand and finger movements with the instrument that produces the acoustic output. The resulting sound is dependent on the detailed interactions of the hands and fingers, specifically in this study, with the piano keys, such as simultaneous positions, velocities, accelerations, and decelerations (Hunt et al., 2000; Hunt and Hermann, 2011). The results of another study on these aspects of pianists’ individuality in the performance of tonal outcomes confirmed that pianists’ abstract notions of timbre correlate with reliable methods of performance technique. These effects also suggest that pianists can express individuality while pursuing a specific interpretive intent (Bernays and Traube, 2014).

### Performance Analysis

#### Historical Background of Piano Roll Sonification

The origins of sonification as a performance score can be traced back to the 1930s, when Carl Seashore’s research pioneered the use of acoustic analysis. To create a performance score that showed the exact variations in timing, pitch, and intensity produced by a musician on an instrument, Seashore invented a piano camera that provided a film and music sample score. It was designed to photographically record the beginning, duration, ending, and relative intensity of notes in a piece. This mechanism is the forerunner of the laser sensors used to measure the depression and release of the hammers on the Disklavier.

Simultaneous auditory and visual sonification enabled sounds that were produced in time and were transient, as opposed to static visual representations, to be repeatedly heard and examined by the viewer. This allowed for comparisons between the models’ performance in terms of technical skill and personal interpretation, and detailed examination of specific elements.

### Findings on Imitation

The process of using auditory-assisted sonification with imitation of model phrases allowed students to see and hear the extent to which they matched the model. It raised awareness of their differences in the perception of dynamics and the actual sound they produced when imitating the models. This also served as a motivation for improvement.

In the first recorded readings of the score, all participants showed errors in the accuracy of the left-hand accompaniment notes. Qualitative responses indicated that visual and auditory attention focused primarily on the right-hand melody in the notated score. The use of auditory support proved to be very effective in enhancing the auditory perception of the sonicated representation of the sounds in both models and their own recorded performances.

**FIGURE 10:**
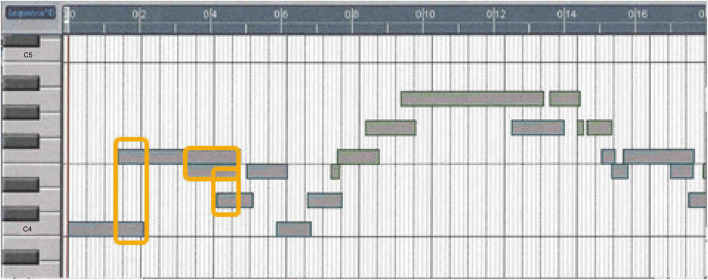
Sonification of note bar overlap in melody, measures 1–6.

The additional feedback on the movement of the keys during Disklavier playback with auditory support provided feedback on the finger technique. Figure 10 shows an original sonification diagram from the research on the heavy overlap of notes.

Before receiving auditory-assisted sonification feedback, this student was unaware of the heavy overlap (Figure 10), indicating poor technical control of note elicitation. Auditory-assisted sonification provided the student with immediate insight into the problem and motivated improvement in the technique.

### Findings on Dynamics

The results showed that more attention was paid to timing than to dynamics in the imitation trials. With repeated practice, the imitations fell within the limits of the dynamic speed levels of the model.

#### Auditory-Assisted Sonification Feedback for Interpretive Choices

In addition, the study examined how this form of feedback can be used to enhance and improve performance. Auditory-assisted sonification became a teaching tool to understand and consider new ways to shape the dynamics and the timing of phrases. The process allowed for accurate identification and articulation of what was heard in terms of timing. Expressive gestures within a time span have meaning in relation to a particular phrase. The performer’s creative realization of the composer’s score was experienced through auditory-assisted sonification.

The greatest difficulty was the perception of dynamic levels. While there was a perceived degree of softness by the students the actual level of sound produced was much greater. The velocity tool in the piano roll editor was used to increase the dynamics of the participants’ own graphs to define the differences necessary to produce the intended sound and motivate experimentation. Playback of each newly played phrase provided feedback on progress.

**FIGURE 11:**
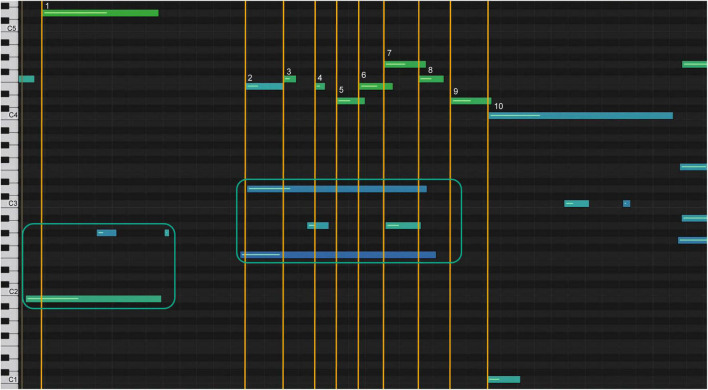
Sonification graph of student’s highpoint before analysis.

Below is an example of how auditory-assisted sonification was used to understand how model C performed a grace note configuration. Figure 11 shows the sonification graph of the student’s first attempt at passage. The yellow lines show the juxtaposition of the right-hand melody with the measures of the left-hand accompaniment notes.

**FIGURE 12:**
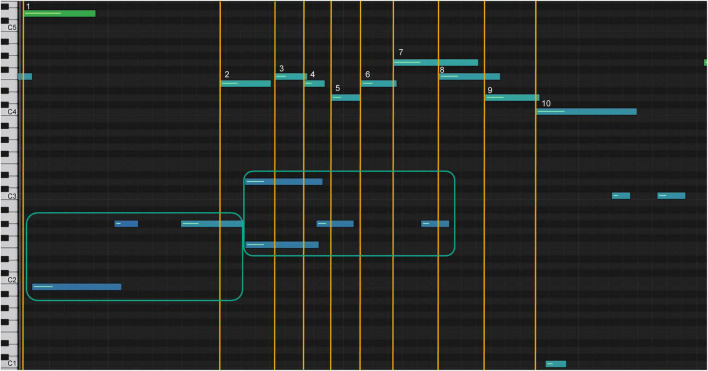
Sonification graph of model C’s highpoint.

The numbered bars represent the melody notes of the passage. The left-hand accompaniment is represented by bars in the teal square. Note 2 of the melody matches the first notes of the accompaniment, note 4 matches the second, and note 7 matches the third (sonification 5). Figure 12 shows the sonification diagram of model C for the same passage (sonification 6). The bars are numbered and marked as above.

Note the marked difference in the spatial representations of the bars. Note 2 occurs before the first notes of the beat in the accompaniment, while the note of the accompaniment is still played from the previous beat. Note 5 occurs after the second note of the accompaniment; the student had played note 4 with the second note of the accompaniment; note 8 occurs after the third note of the accompaniment is played. Notice the stretching of measures 7, 8, and 9, which indicates a slowing of the tempo.

**FIGURE 13:**
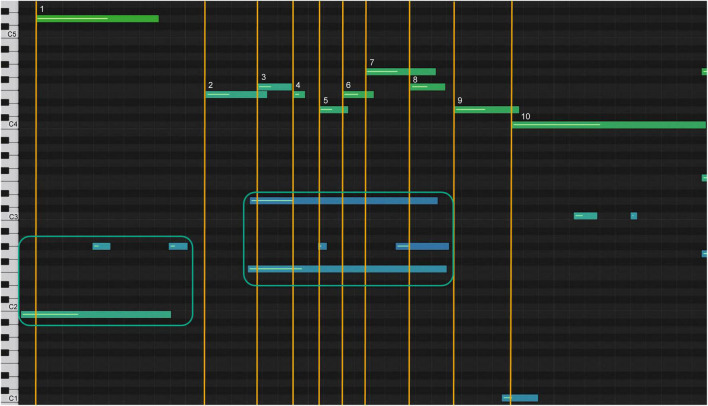
Sonification graph of student’s highpoint after analysis.

After seeing and hearing this sonification, the student understood how the specifics of the timing within the sounds were technically created. Figure 13 shows a diagram of their final rendition of the climax (sonification 7).

The spacing between the note bars of the melody and the accompaniment is marked by the yellow lines, and the lengthening of the intervals between notes 7, 8, and 9 (Figure 13) resembles the model’s shaping of the phrase (Figure 12). This form of sonification represents the lines of performance of a piece of music and draws on the esthetics of tonal music composition (Barrass and Vickers, 2011; Vickers, 2016).

### Participants’ Feedback on Auditory-Assisted Sonification

Students did not listen to tapes or recordings of the music during the week and did not have access to the Disklavier between sessions. One student practiced on an old piano with several defective keys. In all cases, timing and dynamic shaping of phrases were maintained and developed during the week. Tracking the changes in student perception and technical mastery from session to session confirmed the use of auditory feedback as a tool to improve listening comprehension. The students’ qualitative comments confirmed the process.

“The piano roll on the screen helped me hear the differences in the interpretations. Comparing the different color representations of dynamics and length of the bars showed each one’s style” (Student Participant 1).

“The piano roll allowed me to see and understand the subtle differences in the shaping of the phrases while hearing the performances, and to analyze and clearly identify each performer’s interpretive choices” (Student Participant 2).

“I have a crystal-clear image in my mind of the sound from the piano roll graph. The first time I heard only general things–this helped me listen more deeply” (Student Participant 3).

“The graphs helped define for me what I was hearing. This process enabled me to analyze what each performer was doing and gave me a clear picture of what they had in mind” (Student Participant 4).

“Working with the feedback was a valuable learning tool in that one can learn to hear themselves and others in a more objective light” (Student Participant 5).

“Seeing the visual representation along with hearing the playback helped me distinguish the differences in interpretation. Imitating phrases broadened my choices for interpretation. This experience has not only helped me to develop a more critical ear for sound, but it has inspired me to listen more intensely as I create my own interpretation” (Student Participant 6).

“The playback of the performances on the Disklavier along with the performance score on the screen helped me pinpoint my errors. The practice of listening and imitating certain phrases allowed me to consider other possibilities of interpretation” (Student Participant 7).

“Working with the Disklavier and performance score enabled me to critically listen to and compare different pianists’ interpretations. It made it easier to try and imitate them” (Student Participant 8).

Auditory-assisted sonification helped students discover subtle differences in interpretations between performances and enabled them to improve their performance, by listening to the sounds of the symbols on the page and by strengthening the connection in the brain between visual and auditory perception and production. Music interpretation has been referred to as grammar for every performer (Shaffer et al., 1985; Todd, 1985). Auditory-assisted sonification feedback provides a deeper understanding of musical performance of interpretive choices and possibilities.

As an update to this pilot study, one of the participants and one of the artist pianists recently re-recorded their performances with auditory support *via* Zoom. Their comments are as follows:

Visual feedback demonstrated the sound-in-time of the artist’s performance and greatly influenced my auditory perception. Experiencing visual feedback and hearing the performances deepened my awareness of the nuances. This pilot study has had a lasting impact on me both as a musician and as a teacher (Student Participant).

It was powerful to see the rhythmic nuances of my performance precisely spaced out on the graph as I was listening to it. This proves to be extraordinary for teaching. This gave me the experience of seeing sound and space. For students, the simultaneous seeing and hearing of the shapes of the phrases can increase their understanding of the technical approach to the instrument to produce the sounds (Garrick Ohlsson, Concert Pianist).

## Conclusion

Auditory-assisted sonification activated more focused listening and strengthened the link between visual and auditory perception and production. Comparative performance analysis of audio-assisted sonification provided an understanding of the interpretive choices and possibilities and the technical means by which they were produced.

The MIDI in this study provided “a sonification of a sonification,” through which individuals could not only learn the technical aspects of piano playing, but be assisted in moving into a more nuanced appreciation of the music and closer to their essential uniqueness. This can be a catalyst for students to expand their creativity through the subtleties of nuance in expression.

This article should be of interest to a general audience and bring awareness on how MIDI can help further our understanding of teaching and learning expressive music performance through piano pedagogy.

### Limitations of the Study

At the time of writing this research article, the quantitative (numerical) data could not be included because some of the original data files had been lost over time. However, the qualitative results of this exploratory study demonstrated the effectiveness of feedback in teaching the subtleties of interpretation.

The number of participants was not representative of a large group of piano students, or of different levels of proficiency. The experimental design did not include a control group. Furthermore, the participants were not selected through a specific selection process. For future research, specific criteria for participant selection need to be established to assess method effectiveness for students at different developmental levels.

Investigating the long-term effects of auditory/visual feedback was beyond the scope of this exploratory study. However, students demonstrated that they retained the musical insights gained from playing from session to session. One participant recently commented that “this study had a lasting impact on me both as a musician and as a teacher,” which suggests positive long-term retention of musical insights gained from the study.

### Further Research

This exploratory study served as a springboard for further research on how auditory-assisted sonification might be used in teaching piano techniques (Riley and Coons, 2005). Auditory-assisted sonification has been used in conjunction with video and motion analysis software for a more detailed analysis of the piano technique (Riley, 2007). It has also been used in studies using surface electromyography biofeedback (sEMG) for relearning complex technical skills in piano performance (Riley et al., 2005; Riley, 2011).

Future research should include a more comprehensive investigation of auditory-based sonification as a teaching tool through the process of imitation with a larger group of subjects. A control group is needed to compare the effectiveness of sonification with that of traditional teaching methods.

Monitoring the more visceral aspects of emotional and autonomic nervous systems’ involvement by examining this kind of sonification’s effects on the interaction of EEG, EMG, ECG and the recorded sound of the music being performed (to investigate the dynamic interaction of the senses), may provide important insights into the physical, mental, and emotional effects that auditory-assisted sonification has on an individual.

## Data Availability Statement

The original contributions presented in the study are included in the article/Supplementary Material, further inquiries can be directed to the corresponding author.

## Ethics Statement

The studies involving human participants were reviewed and approved by the Office of Sponsored Programs, New York University. The patients/participants provided their written informed consent to participate in this study.

## Author Contributions

The author confirms sole responsibility for the following: study conception and design, data collection, analysis and interpretation of results, and manuscript preparation.

## Conflict of Interest

The author declares that the research was conducted in the absence of any commercial or financial relationships that could be construed as a potential conflict of interest.

## Publisher’s Note

All claims expressed in this article are solely those of the authors and do not necessarily represent those of their affiliated organizations, or those of the publisher, the editors and the reviewers. Any product that may be evaluated in this article, or claim that may be made by its manufacturer, is not guaranteed or endorsed by the publisher.
